# Advancing Cancer Systems Biology: Introducing the Center for the Development of a Virtual Tumor, CViT

**Published:** 2007-03-30

**Authors:** Thomas S. Deisboeck, Le Zhang, Sean Martin

**Affiliations:** 1 Complex Biosystems Modeling Laboratory, Harvard-MIT (HST) Athinoula A. Martinos Center for Biomedical Imaging, Massachusetts General Hospital, Charlestown, MA 02129;; 2 IBM Advanced Internet Technology, Cambridge, MA 02142

**Keywords:** Cancer, complexity, systems biology, multi-scale computational tumor modeling, semantic layered research platform, digital model repository

## Abstract

Integrative cancer biology research relies on a variety of data-driven computational modeling and simulation methods and techniques geared towards gaining new insights into the complexity of biological processes that are of critical importance for cancer research. These include the dynamics of gene-protein interaction networks, the percolation of sub-cellular perturbations across scales and the impact they may have on tumorigenesis in both experiments and clinics. Such innovative ‘systems’ research will greatly benefit from enabling Information Technology that is currently under development, including an online collaborative environment, a Semantic Web based computing platform that hosts data and model repositories as well as high-performance computing access. Here, we present one of the National Cancer Institute’s recently established Integrative Cancer Biology Programs, i.e. the Center for the Development of a Virtual Tumor, CViT, which is charged with building a cancer modeling community, developing the aforementioned enabling technologies and fostering multi-scale cancer modeling and simulation.

## Background

The completion of the Human Genome Project catalyzed a systems view of biomedicine which may well have a dramatic impact on 21st century’s life sciences all together (e.g. Deisboeck T.S. and Kresh J.Y., 2006). The concept, attributed to Aristotle, that *“the whole is more than the sum of its parts”* suggests that dissecting an organism, tissue or cell into ever smaller monomers and hoping to be able to piece it back together afterwards will not work if the underlying mechanisms are anything but linear and involve dynamic relationships. While *systems theory* per se is hardly new (see e.g. seminal work by Bertalanffy v. L., 1968) based on some pioneering yet rather theoretical studies on *complex systems* in biology (e.g. Kauffman S.A., 1993) the notion of *systems biology* as the research approach that integrates biology, medicine, computation and technology to comprehend biological information processing has recently been embraced also by mainstream science (e.g. Ideker et al. 2001; Kitano, 2002). Consequently, this has led to a wave of academic, corporate and governmental efforts in this emerging field. Already at this nascent stage it has become abundantly clear, however, that the transition from classic reductionism driven biomedical science to a systems level understanding of biological processes requires more than access to high-performance computing only. Rather, it needs a new understanding of how multi-scaled biological systems have to be investigated with an approach that integrates computation and experiment and, ultimately, in the case of disease processes, how they can be diagnosed and treated—as systems.

**Figure 1.**
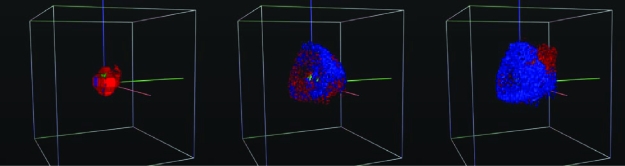
**Multi-Scale Modeling.** 3D snapshots of a virtual brain tumor at three consecutive time points (*left to right*), from Zhang et al. (2007). Blue color represents proliferating tumor cells, while red depicts migratory, green quiescent and grey dead tumor cells. At an early stage the proliferative tumor core appears to be completely surrounded by a cloud of migratory cells, at a later time point, however, a more heterogeneous picture emerges where ultimately a ‘tip’-population of migratory cells can be found adjacent to the location of a nutrient source (*top right quadrant*, not shown).

If a tumor is thought of as a *dynamic self-organizing biosystem*, one can argue that cancer is an almost ideal case to apply the considerable strengths of this new systems biology concept. This is not only because we are still far from deciphering the complexity of all the factors involved in tumorigenesis but also since the countless experimental and clinical studies devoted to it continue to generate an ever growing amount of disparate data with little chance of connecting the ‘dots’ using conventional scientific approaches only. There is no doubt then that innovative computational modeling and simulation, in conjunction with appropriately designed experiments, will rapidly become a valuable if not crucial tool for this new scientific path in cancer biology. Specifically, cutting edge multi-scale computational modeling will be able (a) to help generate experimentally *testable hypotheses*, (b) to *integrate* diverse data, and ultimately, (c) to *predict outcome* also for clinical purposes. Currently, mechanistic dynamical simulations and inferential data mining constitute the two main approaches in interdisciplinary *cancer systems biology* research with significant progress. (1) For instance, molecular pathway simulation has shown promise exemplified through the work by Araujo et al. (2005) who have developed a mathematical model to investigate combination therapy with kinase inhibitors by building upon theoretical studies of the epidermal growth factor receptor (EGFR) pathway (Kholodenko et al. 1999). Another example is Athale et al. (2005, 2006) who, based on previous works by Mansury and Deisboeck (2003, 2004), have modeled a proposed cellular phenotypic switching mechanism also in the EGFR signaling pathway. Most recently, Zhang et al. (2007) have then extended this work in order to simulate the dynamics of EGFR gene-protein interaction profiles, alternating cell phenotypes and emergent multi-cellular patterns with a three dimensional agent-based multi-scaled model (Figure 1). (2) On the other hand, because it is now possible to extract knowledge from large-scale data sets employing advanced data mining techniques (Khalil and Hill, 2005), progress has been made in detecting patterns and correlations in the data that lead to new hypotheses about possible interactions such as on the protein-protein and gene-protein level (e.g. Yeger-Lotem et al. 2004). It is thus a reasonable goal to combine these two promising paths in the future.

Nonetheless, despite, or more accurately, precisely *because* of the significant progress made, several *challenges* for the field have become apparent as well. That is, the absence of a cancer modeling dedicated, collaborative community or network has led to a lack of shared standards or even guidelines let alone unifying platforms to archive, exchange and integrate the many distinct computational and mathematical models that have been and are being developed. This inevitably led to redundancy in some cases where multiple models on for instance ‘cell migration’ have been developed over the years whereas other tumor characteristics have received far less attention. Overall, the result is a diminished impact of the modeling field on experimental cancer research and a loss of potentially valuable time for clinical studies. Therefore, particularly for projects that exceed the capacities of a single team, access to distributed scientific and technical expertise, exchange of knowledge and sharing of biomedical data, modeling algorithms and analysis tools appear to be most critical.

To address these issues and to advance the field of cancer systems biology, the National Cancer Institute, NCI, has recently established the Integrative Cancer Biology Program, ICBP (URL: http://icbp.nci.nih.gov/). One of these nine ICBPs is the **Center for the Development of a Virtual Tumor (CVIT)** at the Massachusetts General Hospital in Boston. In the following section we briefly introduce CViT’s mission and describe the technical infrastructure that it is currently developing in collaboration with IBM’s Advanced Internet Technology.

## Introducing CViT

CViT’s mission is threefold: (1) to establish a **community of investigators** with expertise in the biomedical, the computational and the mathematical aspects of cancer research, and to build the **infrastructure** to enable the group’s sustained growth; (2) to develop a curated **Digital Model Repository** for model archive and exchange; and (3) to advance work towards a **multi-scale modeling platform** *en route* to a modeling tool-kit for cancer research. This section will briefly introduce several major features of CViT, such as the buildup of an online community, the development of the digital model repository and its underlying Semantic Web based research platform, as well as a how CViT envisions to process ‘knowledge integrated’ modeling.

## Building a Cancer Modeling Community: ‘CViT.org’

**Figure 2.**
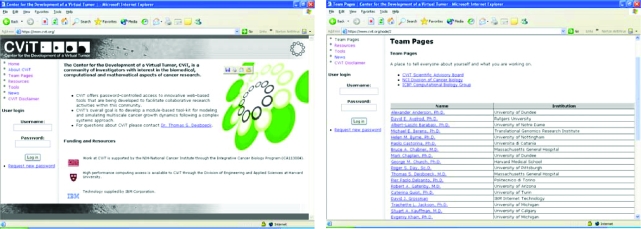
CViT Home Page (*left*) and Investigator Profiles (*right*).

**Figure 3.**
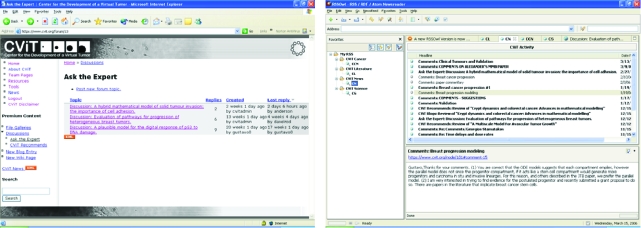
CViT Blog (*left*) and RSS Feeds (*right*).

**Figure 4.**
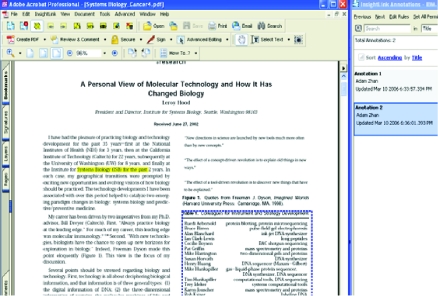
CViT Annotation System.

Cancer research has always been an international enterprise with pockets of critical expertise being developed at numerous sites all over the world and thus now, more so than ever, large-scale consortium projects have to go beyond institutional boundaries to accomplish a set task that would otherwise exceed the resources available at a single site. Given the fact that this entails setup and management of long-distance collaborations that also cross multiple disciplines, a new more flexible collaborative environment has to be created. CViT has built such a user-friendly online platform, CViT. org (URL: http://www.cvit.org) that employs a username & password-protected ‘wiki’-type environment to post and discuss content (Figure 2, *left*). A relatively large portion of the regularly updated information such as researcher profiles (Figure 2, *right*), resources, tutorials and software tools, is already publicly-accessible as part of CViT’s commitment to community outreach. For the continuously growing CViT group of investigators which includes scientists from dozens of institutions around the world, CViT.org provides its participating investigators a number of advanced tools to rapidly communicate, thus facilitating dissemination of knowledge and fostering collaborations: (1) CViT.org offers **“digital notebooks”** or **blogs** such as “Ask the Expert” that have investigators present (on the basis of their PubMedlinked abstracts) one of their recent publications to the group, thereby soliciting feedback and thus setting up a lively discussion forum (Figure 3, *left*). Another blog, “CViT Recommends” is focused on suggestions and recommendations of useful information, including links to up-to-date reviews and upcoming meetings relevant to the field. (2) Secondly, really simple syndication (RSS) is a document specification that gives users the power to collect and organize Web-based news and information in a more efficient manner. **CViT RSS feeds** are intended to encourage CViT members to participate in ongoing discussions within CViT and to facilitate access to and dissemination of cancer modeling-focused, public information. Currently, the site provides news feeds on activity within its own blogs (Figure 3, *right*), on cancer research and, more generally, science news as well as on peer-reviewed literature relevant to cancer modeling efforts. (3) Thirdly, CViT.org broadcasts news, announcements and notifications using its **mail list server** to its membership as well as to the computational biology groups of the entire ICBP. (4) Lastly, an **annotation system** (Figure 4) provided by IBM makes it convenient for CViT’s members to annotate research documents in a variety of formats such as MS Word, PDF and others. This enables capture, collection, management, access and exchange of any such annotations across the distributed teams.

## Knowledge Integrated Modeling and Semantic Layered Research Platform Prototype

CViT’s Digital Model Repository (*below*) will itself be part of a wider application system currently named **Knowledge-Integrate-Modeling (KIM**), which is a custom application designed to support systems biology research. KIM in turn is being constructed on IBM’s prototype **Semantic Layered Research Platform (SLRP)**. The Semantic Layered Research Platform is primarily based on GRID and Semantic Web^1^ technologies and concepts (Neumann, 2005). In particular SLRP provides a central database for data represented in the **Resource Description Framework (RDF)**^2^, a highly flexible method of representing data conforming to practically any schema. All data and system objects are named using **Life Science Identifiers (LSID’s)**^3^ an industry standard for uniquely naming and thereby identifying any digital artifact. LSID’s provide a handle by which that object may be later retrieved and a unique key by which meta-data about that object or its relationship to other objects may be attached or retrieved. GRID technologies provide a systems management framework for the automatic deployment and tending of software systems and applications for compute and data intensive tasks in large clusters of computers sometimes deployed over a wide area. Multi-scale modeling (i.e. combining molecular, microscopic and macroscopic data levels) is such a potentially very data intense yet most promising tool for cancer systems biology (for an example, see Figure 1). CViT not only will support this type of approach through KIM but also will deposit such algorithms and their simulation results in the Digital Model Repository (see below).

The **SLRP platform** will provide application services to: (1) *Connect* a CViT researcher’s desktop directly to the powerful databases in which all research information collected by a collaborative research effort is held along with its associated semantic linkages to other data items as well as the meta-data that provides each piece of stored information with a context. (2) Enable *wide area collaborations* as characteristic for CViT by allowing the sharing of any data object in the system along with its semantic context, linkages and meta-data, thus allowing an interconnected web of information to easily be communicated between collaborators. (3) Allow the *structured annotation* of any object in the system, such as an area of a microscopy or MR-image, or a parameter in an experimental result, whether it be to add additional meta-data or simply to note a semantic relationship between stored objects. (4) Provide interfaces to the *GRID compute platform* where models or digital experiments can be executed by the assembly and execution of workflows representing digital experiments by end-user researchers, without the need for a thorough technical understanding of the sophisticated computer systems involved. (5) Provide a *visual interface* for the integrated viewing and annotating of all information associated with and created by a research effort. (6) Provide a collaborative environment designed for the *creation, debugging & maintenance of the source code* modules associated with a research effort that requires significant computer aided modeling, simulation and data intensive computing. The latter is of particular importance for the DMR.

**Figure 5.**
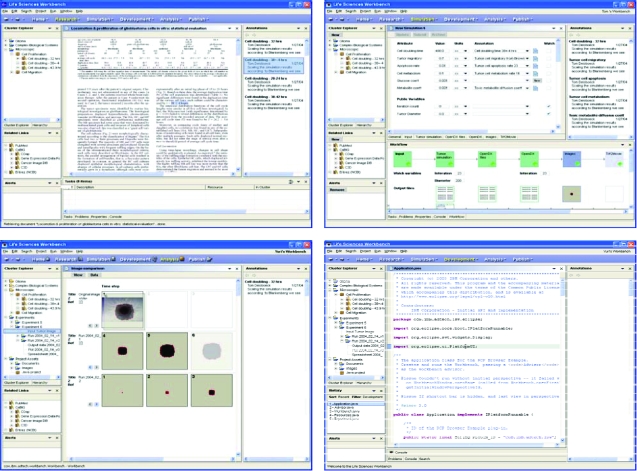
**Knowledge Integrated Modeling**. Employing SLRP annotated data (e.g. from PubMed listed manuscripts; *top left*) are captured and used as model input parameters (*top right*). Deploying the code (language editing perspective, *bottom right*) to a high-performance compute environment yields *in silico* results that can then be compared with experimental data such as the microscopy images of tumors cultured *in vitro* (*bottom left*), or back to data published in the literature. This feedback allows continuous refinement of the (DMR-archived) algorithm(s) and spurs design and development of experiments.

CViT researchers using the KIM application will be provided with a “Biologists Workbench” application program that can be customized to aid in the tasks that scientist carries out in his/her day to day work and collaborations with colleagues. This application will be based on the **Eclipse Framework** (URL: http://www.eclipse.org), an open source, cross-platform, Integrated Development Environment that is now also being used to create regular end-user applications. The Eclipse Framework already provides a great deal of support for code developers including source code editors, source code repository integration, viewers and debuggers. Through the addition of SLRP components for annotation and connectivity to the central SLRP data stores and custom Eclipse Perspectives (a perspective is a mechanism for extending the Eclipse visual framework) for tasks like biomedical image manipulation and comparison, wide-area collaboration, views for clustering of related data objects or editing information in PDF, spreadsheet, word processing documents or web formats, and for creating or reviewing digital experiment work flow assemblers, this environment will provide an excellent single desktop work space for the CViT community (Figure 5).

## The Digital Model Repository (DMR)

The Digital Model Repository will utilize the **Concurrent Versioning System (CVS;** URL: http://ximbiot.com/cvs/wiki/index.php) as the primary source code control system. CVS enjoys wide support from development tooling in general and the Eclipse IDE in particular. It will provide the means by which CViT developers will collaborate, track, control and backup the source code for their evolving models over a long period of time. The ViewCVS open source system or something similar will be used to provide a Worldwide-web browser based interface into the DMR along with the direct CVS connections over the internet. The system will include all the appropriate security mechanisms to secure access to the source code system for either reading or writing as appropriate to the collaboration. In addition to CVS, the DMR will establish obvious Life Science Identifier (LSID) name mappings to all source code files and modules. A LSID Resolver (Clark et al. 2004; Martin et al. 2005) will be established for the DMR. Every version of every source code file and module in the DMR will always have a unique name in its own LSID. The combination of CVS source code control and LSID technologies will allow CViT model developers to have a means (the LSID name) to know exactly which source code file version and supporting code libraries were used for any particular model execution run. When necessary they will be able to use this unique identifier to annotate, comment, or make unambiguous reference to these code modules in emails or research papers.

**Figure 6.**
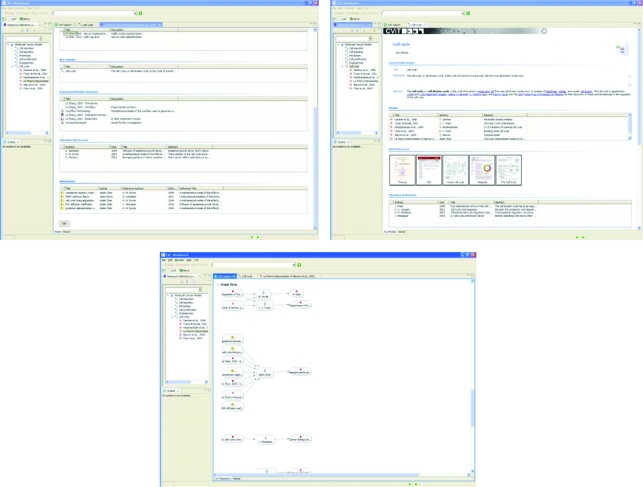
**Digital Model Repository (DMR)**. Shown are three ‘Biologist Workbench’ views of data in the DMR. The first image (*top left*) depicts an actual *model instance*, with links to the model artifacts, experimental data, source code, abstract representation (equations), related literature, annotation and, of course, the results, which may include biomedical images, video-microscopy and computer simulation movie. The second image (*top right*) shows how a user may choose to view the *key module* to which the model belongs whereas the third image (*bottom*) visually describes the *semantic inter-relationship* links between data elements in series of models.

We note in this context that any model description consists not only of the algorithm, its code implementation and, if available, its markup language representation (see below), but also of, for instance, related experimental data, simulation results, visualizations and manuscripts (Figure 6). Researchers using the SLRP system to track their modeling will automatically create and store semantic links to the inputs and output of any particular model run, along with LSID references to the actual source code modules that were executed for any particular simulation execution run. The system will automatically document and make directly accessible the knowledge required to exactly reproduce a digital experiment without fear that the an incorrect version of the source code or input parameter data is being used.

Usage of CViT’s DMR content by participating investigators and their institutions will be enabled through an already posted open source license that is approved by the National Cancer Institute and in compliance with guidelines put forward by the Cancer Biomedical Informatics Grid, caBIG. Close interaction with caBIG is planned and will likely include caBIG standards compliant web-service to both use and provide data and access to high-performance computing as well as specialized analysis algorithms. Finally, more work may be required at a later stage on the representation level if the models in CViT’s repository are to be integrated with those in other *non-cancer* focused digital repositories. Examples for such model representations include CellML (URL: http://www.cellml.org/; its repository can be accessed at URL: http://www.cellml.org/models) which has already found use in the larger biomedical modeling community. Since CellML includes MathML, equations are straightforward to add. Additionally, the RDF section of CellML appears to be adaptable to describe other model parameters and would potentially integrate well with CViT’s overall underlying system platform that is based on RDF as mentioned earlier. Another alternative is SBML (URL:http://sbml.org/index.psp; used e.g. by the repository of the European Bioinformatics Institute (URL:http://www.ebi.ac.uk/biomodels/)), which, although focused primarily on the biochemical pathway level, has however more libraries and parsers available and has been implemented in many biological modeling tools.

## Conclusion

The new paradigm of systems biology holds great promise for progress, from biomedical basic science (e.g. Kitano, 2002) to drug discovery (e.g. Hood and Perlmutter, 2004), and therefore in particular for cancer research (Coffey, 1998; Hornberg et al. 2006; Khalil and Hill, 2005; Waliszewski et al. 1998). Integrative cancer systems biology is built on the premise that a better understanding of the complexity of tumorigenesis requires design, development and implementation of novel, data-driven *in silico* models that account for the multi-scaled processes involved. However, cancer modeling algorithms are usually developed in a non-standardized fashion, often very specific in addressing a particular problem of interest, thus commonly lack widespread distribution, therefore acceptance in and feedback from experimentalists and clinicians. While one must concede that currently there is no perfect cancer model available or in sight, this fact all the more emphasizes the need of sharing and comparing available models as well as the data that went into them or are derived from them, thus for archiving algorithms just as much as for exchanging concepts and results. It is here where CViT will make a difference. Its growing international group of investigators not only represents already an unparalleled level of expertise, it also allows for rapid dissemination of information relevant to the field using the tools made available at CViT.org. The technologies CViT currently develops, specifically its semantic layered research platform with its digital model repository and knowledge integrated modeling workflow address critical needs of the community and thus undoubtedly will help advance the field of integrative cancer biology also beyond the current scope of NCI’s ICB program. All this creates a cutting edge environment that can make CViT’s long term vision for multi-scale cancer systems biology research a reality: i.e. the development a *module-based cancer modeling tool-kit* that can be specified as need be in support of *personalized medicine*.

## Footnotes

1 Berners-Lee,T., Hendler, J., Lassila, O. (2001) The Semantic Web, Scientific American, May 2001 (URL: http://www.scientificamerican.com/article.cfm?articleID=00048144-10D2-1C70-84A9809EC588EF21&catID=2).

2 Miller E, Swick R, Brickley D (2003) ‘W3C Resource Description Framework (RDF)’ (URL:http://www.w3c.org/RDF/).

3 EBI, I3C, IBM (2003) ‘Life Science Identifiers RFP Response Revised Joint Submission’, Object Management Group Document lifesci/03-10-01 (URL: http://www.omg.org/cgi-bin/doc?lifesci/2003-10-01).
